# Endometriosis Patients in the Postmenopausal Period: Pre- and Postmenopausal Factors Influencing Postmenopausal Health

**DOI:** 10.1155/2014/746705

**Published:** 2014-06-02

**Authors:** Dietmar Haas, Peter Wurm, Wolfgang Schimetta, Kathrin Schabetsberger, Andreas Shamiyeh, Peter Oppelt, Helge Binder

**Affiliations:** ^1^Department of Obstetrics and Gynecology, Women's General Hospital, Krankenhausstraße 26–30, 4021 Linz, Austria; ^2^Department of Gynecology, Erlangen University Hospital, Universitätsstraße 21–23, 91054 Erlangen, Germany; ^3^Faculty of Medicine, Johannes Kepler Univerity, Altenberger Straße 69, 4040 Linz, Austria; ^4^Department of Applied Systems Research and Statistics, Johannes Kepler University, Altenberger Straße 69, 4040 Linz, Austria; ^5^Ludwig Boltzmann Institute for Operative Laparoscopy, Second Surgical Department, Linz General Hospital, Krankenhausstraße 9, 4021 Linz, Austria; ^6^Department of Obstetrics and Gynecology, Cantonal Hospital Uri, Spitalstraße 1, 6460 Altdorf, Switzerland

## Abstract

*Objective*. To evaluate patients' health status and the course of endometriosis from the premenopausal to the postmenopausal period and evaluate influencing factors that may be relevant. *Methods*. Questionnaire completed by 35 postmenopausal women in whom endometriosis had been histologically confirmed premenopausally. Correlation and regression analyses were carried out to identify factors relevant to their postmenopausal health status. *Results*. Overall, there was clear improvement in typical endometriosis symptoms and sexual life. Clear associations (*P* < 0.005) were observed between premenopausal factors like physical limitations caused by the disease, impaired social contacts and psychological problems, and postmenopausal pain and impairment of sexual life. Three statistical models for assessing pain and impairment of sexual life in the postmenopausal period were calculated on the basis of clinical symptoms in the premenopausal period, with a very high degree of accuracy (*P* < 0.001; *R* ^2^ = 0.833/0.857/0.931). *Conclusions*. The results of the survey strongly suggest that physical fitness and freedom from physical restrictions, a good social environment, and psychological care in both the premenopausal and postmenopausal periods lead to marked improvements in the postmenopausal period with regard to pain, dyspareunia, and influence on sexual life in endometriosis patients.

## 1. Introduction

Endometriosis is one of the most common gynecological diseases, with an estimated current incidence of 40,000 new patients per year in Germany [1]. Worldwide, nearly 80 million women are affected by the disease. Data in the medical literature suggest that the prevalence of endometriosis is 10–15% in all women of reproductive age [1–3].

Several theories on the etiology and pathogenesis of endometriosis have been proposed, but a definitive explanation of the pathophysiological mechanism involved has not yet been found. Three basic theories are under discussion: the theory of cell transplantation [4], the theory of metaplasia [5], and the theory of the endometrial-subendometrial unit or “archimetra” [6]. Immunological, endocrinological, genetic, and inflammatory factors also appear to be essential elements in the pathogenesis of endometriosis [7–14]. However, estrogen dependence is considered to be central to the pathophysiological process and persistence of the lesions [11, 15]. The latter concept has led to the widely held belief that endometriosis is a disease of premenopausal women that is “cured” by the menopause [16, 17].

Cases have occasionally been reported in the literature describing endometriosis in the postmenopausal period in patients with or without hormone replacement therapy [18–24]. However, the current state of the data is inadequate to allow any assessment of this and the mechanisms underlying the entity have not been explained [25].

The aim of the present study was to evaluate health status and the course of endometriosis from the premenopausal to the postmenopausal periods. In addition, relevant factors influencing this were to be identified. Using a statistical model, an attempt was made to calculate the expected health status in the postmenopausal period on the basis of premenopausal clinical symptoms.

## 2. Methods

Institutional review board (IRB) approval was obtained (ref. number K-20-12). Data for 35 endometriosis patients who were postmenopausal at the time of responding to a questionnaire were collected and statistically analyzed in this epidemiological study. Before the menopause, all of the participants had undergone surgery for endometriosis, with the findings confirmed histologically.

The inclusion criteria were histologically confirmed: premenopausal endometriosis and age ≥55 years, with the last menstruation being at least 12 months previously. Patients with bilateral adnexectomy who were not receiving hormone replacement therapy were also included. Exclusion criteria were questionnaires that were not fully completed and patients under the age of 55 who had undergone hysterectomy premenopausally. The hysterectomy would have made it impossible to obtain a menstrual history, giving rise to bias in relation to menopausal status.

A total of 150 questionnaires were presented to two self-help groups (the Austrian Endometriosis Association and the German Endometriosis Association) and were also made available in our own outpatient gynecological department. Forty-one women decided to participate in the study anonymously. The anonymous questionnaire letter boxes were opened at the end of 6 months and the forms were checked for completeness. Six of the 41 questionnaires had not been fully completed or did not match the inclusion criteria. All of the questions were explicitly related to endometriosis. The patients were thus instructed to respond to the questions—for example, in relation to “psychological problems”—exclusively in relation to endometriosis. As an alternative response, the patients were also given the option “due to a different cause.”

The questionnaire included a total of 147 questions, divided into three parts. Part 1 (29 questions) was concerned with the patient's general medical history (18 questions), including social and family history and also surgical history (11 questions). Part 2 (54 questions) inquired into symptoms (21 questions) and complaints (33 questions) in the period before the menopause. Part 3 (64 questions) was concerned exclusively with questions about symptoms (24 questions) and complaints (40 questions) in the postmenopausal period. A visual analogue scale (best grade: 0, poorest grade: 10) was used for responses to questions about pain and impairment of sexual life (best grade: 0, poorest grade: 10).

### 2.1. Statistical Analysis

The exact Wilcoxon test was used to compare the patients' general condition before and after the menopause. Spearman's rank correlation coefficients and point biserial correlation coefficients were calculated to assess correlations. Multiple stepwise regression analyses were used to investigate the predictability of the intensity of general pain in the postmenopausal period (0–10), pain intensity during sexual intercourse in the postmenopausal period (0–10), and the influence of endometriosis on sexual life in the postmenopausal period (0–10) relative to variables from the premenopausal period (all of these independent variables are listed in Supplemental Digital Content 1 available online at http://dx.doi.org/10.1155/2014/746705). Type 1 error was not adjusted for multiple testing, and all *P* values presented are therefore only descriptive. The open-source *R* statistical software package, version 2.14.1 (Institute for Statistics and Mathematics, University of Vienna, Austria), was used for statistical analysis.

## 3. Results

The group of patients consisted of 35 women aged 37–79 years. The patients' average age was 53.9 ± 9.78 years. Their average age at the onset of menopause was 43.03 years. The median time from menopause to completing the questionnaire was 11 years (Table 1).

Each participant had undergone a mean of 2.74 ± 1.69 gynecological operations due to endometriosis at the time of the questionnaire.

All of the patients (100%) stated that they were enjoying or had enjoyed their occupations, including nine (25.7%) who were retired at the time of the questionnaire.

To the question of how often the participants had been pregnant, nine (26%) responded that they had never been pregnant. Ten (28%) had been pregnant once, nine (26%) twice, and seven (20%) more than twice. Nine participants (26%) had not given birth to any children, 13 (37%) had given birth once, 11 (31%) had had two children, and two (6%) had given birth to more than two children.

There were no relevant differences from the normal population with regard to concomitant diseases. The large number of 21 patients with allergies (60%) was notable. Six participants (17.1%) stated that they were regular smokers at the time of the questionnaire, while 16 (45.7%) had been smokers in the past.

With regard to family history, nine patients (25.7%) stated that their mothers had had dysmenorrhea; three of the mothers had histologically confirmed endometriosis. Six patients (17.1%) reported that a sister had dysmenorrhea; five of the six sisters had histologically confirmed endometriosis. Eight patients (22.9%) stated that their daughters had dysmenorrhea; three of the daughters had histologically confirmed endometriosis.

### 3.1. General Health Status

**Figure 1:**
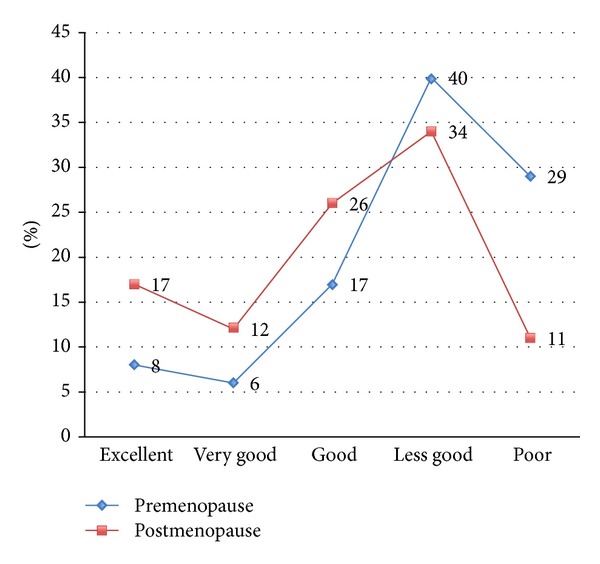
Comparison of the general condition of endometriosis patients in the premenopausal and postmenopausal periods.

Eleven patients (31.4%) described their general state of health in the premenopausal period as “excellent” to “good,” while 24 patients (68.6%) described it as “not so good” to “poor.” In the postmenopausal period, 18 patients (51.4%) described their general state of health as “excellent” to “good,” while 17 patients (48.6%) described it as “not so good” to “poor” (Figure 1).

When comparing their premenopausal and postmenopausal status, in both, 26 of the 35 patients (74.3%, resp.) stated that they had limitations in strenuous physical activities, while 25 versus 23 (71.4% versus 68.6%) stated that they had limitations in moderate physical activities. Impairment was experienced by 21 versus 20 patients (60% versus 57.1%) when carrying a shopping bag, by 19 versus 17 (54.3% versus 48.6%) when climbing several steps, and by seven versus nine (20% versus 25.7%) when climbing a single step. Impairment when “bending/kneeling/stooping” was experienced by 19 versus 18 of the patients (54.3% versus 51.4%) and difficulty in walking approximately 1 km by 18 versus 12 of the patients (51.4% versus 34%). Whereas 62.9% of the patients attributed these restrictions to endometriosis in the premenopausal period, 22.9% of them attributed their physical limitations to endometriosis in the postmenopausal period.

In the premenopausal period, psychological problems were reported by 51.4% of the patients and restriction of social contacts by 62.9%, and as many as 80% described occupational restrictions due to endometriosis. The corresponding postmenopausal figures were 20%, 17.1%, and 20%.

### 3.2. Pain History

Thirty-two patients (91.4%) had had dysmenorrhea since the menarche, with a median intensity of 7.0. Thirty-three patients (94.3%) reported abdominal pain in the premenopausal period, with a median pain intensity of 8.0. As many as 22 patients (62.9%) reported pain in the postmenopausal period, with a median of 3.0. These patients all attributed the pain to the endometriosis. Seven of the 22 patients also reported pain due to other causes.

Twenty-five of the patients (71.4%) had already had dyspareunia premenopausally, and it persisted postmenopausally in 19 patients (54.3%). The median values for its intensity were 5.0 and 2.0, respectively.

Twenty-eight patients (80%) reported that their sexual life had been affected by the endometriosis in the premenopausal period, and the figure was still as high as 19 patients (54.3%) in the postmenopausal period. The median values for this effect were 5.0 and 1.0 (Table 1). Other symptoms the patients experienced are summed up in Table 2.

Premenopausally, 21 patients (60%) had regularly taken analgetics; 10 (28.6%) had taken gonadotropin-releasing hormone (GnRH) analogues; and 15 patients (42.9%) had taken the contraceptive pill. The mean for the total period of drug intake was 40.9 months. Twenty-three of the patients (65.7%) stated that taking medication had not led to any improvement in symptoms, and side effects developed in 37.1%.

A total of 26 patients (74.3%) had undergone hysterectomy, 10 of them with bilateral adnexectomies. Three patients had a bilateral adnexectomy without hysterectomy. At least one laparoscopy was carried out on 32 patients and 16 patients had at least one laparotomy. Thus, 13 patients had at least one laparoscopy and a laparotomy.

### 3.3. Correlation Analyses

Table 3 shows the most notable associations between the general intensity of pain, pain intensity during sexual intercourse, and influence on sexual life in the postmenopausal period, on the one hand, and various premenopausal and postmenopausal variables, on the other hand. All of the parameters are listed in Supplemental Digital Content 1. Factors that correlated poorly with the target variables were concomitant diseases and bowel symptoms, family history, all forms of drug intake including the period of medication and alternative therapies, pregnancies, and parity. In addition, only a slight association was noted between the number, type, and method (surgical technique) of operations and postmenopausal target variables, with the exception of hysterectomy and adnexectomy or combinations of them (Table 3). There were no correlations worth mentioning between bladder symptoms in the premenopausal period and those in the postmenopausal period.

### 3.4. Regression Analyses

Multiple stepwise regression analyses resulted in statistical models with remarkably high levels of predictive power and accuracy for predicting the general intensity of pain in the postmenopausal period (*P* < 0.001; *R* ^2^ = 0.833), pain intensity during sexual intercourse in the postmenopausal period (*P* < 0.001; *R* ^2^ = 0.857), and influence on sexual life in the postmenopausal period (*P* < 0.001; *R* ^2^ = 0.931) on the basis of information available premenopausally (for details, see Supplemental Digital Content 2).

## 4. Discussion

It is interesting that concomitant diseases and bowel symptoms (Table 2), family history, all forms of medication including their duration and alternative therapies, pregnancy, and parity proved to be quite unimportant influencing factors relative to the postmenopausal target variables mentioned. The number, type, and method (surgical techniques) of operations carried out also hardly correlated at all with the target variables “general pain experienced,” “pain during sexual intercourse,” and “disturbance of sexual life,” with the exception of hysterectomy and adnexectomy and the combinations of them listed in Table 3. It is notable here that hysterectomy with the adnexa or bilateral adnexectomy led to marked deterioration of symptoms during the postmenopausal period.

As Figure 1 shows, there was a clear improvement in the patients' general condition when the premenopausal and postmenopausal periods are compared. However, it should be pointed out that this parameter is probably composed of several factors. It appears that a poor general state of health during the premenopausal period markedly correlates with more severe general pain in the postmenopausal period (Table 3).

Clear improvement with regard to pain, dyspareunia, and influence on sexual life is seen in the postmenopausal period (Table 1). However, it is also notable here that general pain and its intensity in the premenopausal period are not significantly associated with any postmenopausal findings. By contrast, dyspareunia and influence on sexual life in the premenopausal period certainly correlate well with symptoms in the postmenopausal period (Table 3).

We would interpret these results as follows. As endometriosis is a long-term disease that usually has a course lasting several years, the symptoms and complaints in the premenopausal period can become chronic and can thus have an effect on postmenopausal life [26]. Dyspareunia and a negative effect on sexual life in the premenopausal period may thus perhaps be able to leave “inner scars” that have negative effects on the postmenopausal period. On this view, general pain that is not necessarily associated with sexual life appears to have a less marked effect on the period after the menopause.

The results with regard to psychological problems, impairment of social contacts, and impairment of everyday life and working life due to endometriosis in the premenopausal period are surprising. Effects of these are seen very clearly during the postmenopausal period in the deterioration in general pain experienced, pain during sexual intercourse, and disturbances of sexual life. There is also evidence in the literature in this connection showing that chronic pelvic pain (CPP) can have a negative influence on family life, sexual life, and social life [26]. The target variables in the postmenopausal period understandably also deteriorate from the psychological point of view (Table 3). The patients were asked to relate psychological problems, impairment of social contacts, and impairments of everyday life and working life only to the endometriosis. It might be questionable whether it is really possible for patients to assign such psychological factors to endometriosis in isolation and objectively. However, the data suggest that the patients were in fact able to do this, since a marked decline in these factors was observed in the postmenopausal period.

Similarly surprising were the results with regard to physical restrictions (Table 3). Almost all physical restrictions in the premenopausal and postmenopausal periods correlate very strongly with the postmenopausal target variables (general pain experienced, pain during sexual intercourse, and disturbances of sexual life). This clearly shows how important maintenance of physical fitness is even in the premenopausal period.

Stress plays a very important role in the clinical picture of endometriosis [27, 28]. It has been shown in an animal model that stress leads to a deterioration in endometriosis [29]. It has been well demonstrated that stress is often linked to nicotine consumption [30]. This is also reflected in the present study, in which the proportion of smokers was notably high (46%) in comparison with that in the general population in Austria (19% of women over the age of 15) [31].

There was also a high proportion of patients with allergies (60%). Comparable prevalence figures among women in the general population are 25% in Austria [32] and 29% in Switzerland [33]. Studies have shown that endometriosis patients suffer significantly more often from immunodeficiencies, asthma, and allergies [34]. This might also be attributable to increased stress caused by the endometriosis, leading to a deterioration in the immune system [35, 36]. However, it has already been clearly shown that physical exercise and psychological care lead to a marked reduction in the level of stress in endometriosis patients [37].

On the basis of the present results, it can be concluded that physical fitness and an absence of physical symptoms, a good social environment, and psychological care not only during the premenopausal period but also in the postmenopausal period as well lead to marked improvement with regard to pain, dyspareunia, and effects on sexual life. In Germany, there are already two rehabilitation centers for endometriosis patients that have been certified by the Endometriosis Research Foundation (*Stiftung Endometriose-Forschung* (SEF)) [38]. The centers focus on physical exercise, physiotherapy, and psychological care.

If the results of the present study are confirmed by further research, there will be an urgent need for accessible institutions of this type to be established in every country in the world. This is particularly the case in view of the fact that endometriosis has been identified as a high cost factor in studies investigating economic targets, which have indicated that a potential cause of this might be inadequate infrastructure [39–43].

The good predictability of the target parameters, “general intensity of pain in the postmenopausal period,” “pain intensity during sexual intercourse in the postmenopausal period,” and “influence on sexual life in the postmenopausal period” relative to premenopausal factors, might be due to endometriosis patients' very good ability to recall the symptoms and complaints that they had during the premenopausal period (Supplemental Digital Content 2).

Despite the high level of statistical accuracy of the models, the current state of knowledge and the questionable representativeness of the data discussed above do not make it currently possible to draw any detailed conclusions regarding the actual course of the disease. Therapeutic decisions should on no account be made on the basis of these models. Endometriosis in itself represents an extremely complex and polymorphous disease, and it is influenced by the patients' individual characters. The absolute focus should always be on individual consideration and treatment of each and every patient.

A major limitation of the present study is the relatively small number of cases included. This is due to the difficulty of accessing a relevant group of patients with treatments that may already lie decades in the past. In addition, the fact that evidently only a small proportion of candidate patients decided to contribute their information and complete the questionnaire substantially increases the chances that individuals with exceptionally positive or exceptionally negative experiences may be overrepresented (recall bias). Furthermore, the choice of a self-help group for the investigation increases the risk of a selection bias. However, irrespective of this limitation on the validity of the study, it would be valuable to take the very marked and partly surprising results of this survey as an interesting approach that needs to be investigated in further research.

## 5. Conclusions

Physical fitness and freedom from physical symptoms, a good social environment, and psychological care not only in the premenopausal period but also in the postmenopausal period lead to a marked improvement with regard to pain, dyspareunia, and effects on sexual life during the postmenopausal period in patients with endometriosis. Rehabilitation clinics for endometriosis patients, aimed at maintaining their physical fitness and providing psychological care, should be available and accessible. In view of the patients' good memory of their complaints and symptoms during the premenopausal period, it is possible to anticipate complaints and their severity in the postmenopausal period relatively precisely using these premenopausal factors alone—and with this information, it may be possible to influence them in a positive way.

## Supplementary Material

In supplemental digital content 1 all variables of the quetionnaire and their correlation coefficients for the three target variables “Pain intensity”, “Dyspareunia” and “Sexual dysfunction” in the postmenopausal period are listed.
In supplemental digital content 2 all questionnaire items are listed, which were used as independent variables for all multiple regression analyses. All of the independent variables were related only to the premenopausal, active period of endometriosis. Furthermore, the best three regression models for predicting an effect on general postmenopausal pain, postmenopausal pain during sexual intercourse and impairment of postmenopausal sexuality due to premenopausal factors are provided.

## Figures and Tables

**Table 1:** Patients (*n* = 35).

	Mean ± SD
Postmenopausal period (in years)	10.8 ± 11.14
Pain intensity since menarche (0–10)	6.3 ± 2.85
Premenopausal abdominal pain (0–10)	7.2 ± 2.81
Postmenopausal abdominal pain (0–10)	3.3 ± 3.40
Premenopausal dyspareunia (0–10)	4.1 ± 2.94
Postmenopausal dyspareunia (0–10)	2.3 ± 2.87
Operations (numbers)	2.7 ± 1.69
Premenopausal effect on sexual life (0–10)	5.3 ± 3.65
Postmenopausal effect on sexual life (0–10)	2.8 ± 3.73

**Table 2:** Symptoms.

Symptoms	Premenopausal (*n*, %)	Postmenopausal (*n*, %)
Intestinal cramp	21 (60%)	11 (31.4%)
Dyschezia	18 (51.4%)	9 (25.7%)
Diarrhea	11 (31.4%)	9 (25.7%)
Constipation	14 (40%)	10 (28.6%)
Mucus in feces	16 (45.7%)	6 (17.1%)
Blood in feces	8 (22.9%)	3 (8.6%)
Dysuria	11 (31.4%)	10 (28.6%)
Hematuria	6 (17.1%)	0 (0%)
Increased urinary urgency	15 (42.9%)	12 (34.3%)
Incontinence	13 (37.1%)	18 (51.4%)
Retrosymphyseal pain	10 (28.6%)	8 (22.9%)

**Table 3:** Correlation coefficients for associations between parameters for the patient's medical history and health status, on the one hand, and postmenopausal impairment of sexual life and pain intensity, on the other hand.

	General intensity of pain	Pain intensity during sexual intercourse	Impairment of sexual life
**General medical history**			
Allergies	0.386*	0.171	0.210
Hysterectomy without adnexa	−0.335*	−0.284	−0.271
Hysterectomy with bilateral adnexectomy	0.412*	0.447**	0.445**
Bilateral adnexectomy	0.258	0.425*	0.424*
**Premenopausal period**			
General pain	0.276	0.135	0.244
General pain intensity	−0.022	0.228	0.226
Pain during sexual intercourse	0.284	0.354*	0.287
Pain intensity during sexual intercourse	0.370*	0.385*	0.287
Effect of endometriosis on sexual life	0.385*	0.625**	0.607**
Poor general condition	0.483**	0.248	0.244
Physical restriction due to endometriosis	0.500**	0.427*	0.336*
Physical restrictions during			
Strenuous activities	0.460**	0.359*	0.300
Moderately strenuous activities	0.516**	0.410*	0.287
Carrying shopping bag	0.461**	0.366*	0.341*
Climbing steps	0.453**	0.564**	0.362*
Climbing one step	0.353*	0.359*	0.306
Bending/kneeling/stooping	0.643**	0.588**	0.401*
Walking 1 km	0.548**	0.496**	0.385*
Bathing/dressing	0.256	0.260	0.089
Psychological problems	0.530**	0.514**	0.453**
Impairment of social contacts	0.500**	0.507**	0.506**
Impairment of everyday living/work	0.182	0.433**	0.496**
**Postmenopausal period**			
Pain during urination	0.409*	0.443**	0.482**
Increased urinary urgency	0.396*	0.438**	0.367*
Physical restriction due to endometriosis	0.676**	0.691**	0.605**
Physical restrictions during			
Strenuous activities	0.553**	0.499**	0.402*
Moderately strenuous activities	0.596**	0.512**	0.501**
Carrying shopping bag	0.553**	0.396*	0.467**
Climbing steps	0.606**	0.389*	0.170
Climbing one step	0.516**	0.376*	0.293
Bending/kneeling/stooping	0.658**	0.643**	0.456**
Walking 1 km	0.644**	0.441**	0.349*
Bathing/dressing	0.406*	0.092	0.031
Psychological problems	0.471**	0.435**	0.407*
Impairment of everyday living/work	0.706**	0.459**	0.548**

*P < 0.05.

**P < 0.01.
