# Supplementary material for: Endometriosis Patients in the Postmenopausal Period: Pre- and Postmenopausal Factors Influencing Postmenopausal Health
Source: Biomed Res Int. 2014 Jun 2;2014:746705. doi: 10.1155/2014/746705 (PMC4058668; doi:10.1155/2014/746705)
Supplement: Supplementary file 1 — In supplemental digital content 1 all variables of the quetionnaire and their correlation coefficients for the three target variables “Pain intensity”, “Dyspareunia” and “Sexual dysfunction” in the postmenopausal period are listed. In supplemental digital content 2 all questionnaire items are listed, which were used as independent variables for all multiple regression analyses. All of the independent variables were related only to the premenopausal, active period of endometriosis. Furthermore, the best three regression models for predicting an effect on general postmenopausal pain, postmenopausal pain during sexual intercourse and impairment of postmenopausal sexuality due to premenopausal factors are provided. [file 746705.f1.pdf]

SUPPLEMENTAL DIGITAL CONTENT 1:

Correlation coefficients for the three target variables in the postmenopausal period.

	**Postmenopausal**		
	**Pain intensity**	**Dyspareunia**	**Sexual dysfunction**
**General**			
Pain intensity *post*		0.540**	0.465**
Dyspareunia *post*	0.540**		0.875**
Sexual dysfunction *post*	0.465**	0.875**	
Age	–0.133	–0.194	–0.167
Retired (yes/no)	0.200	–0.041	–0.058
Hospital stays	0.371*	0.353*	0.372*
Hypertension	0.177	–0.030	–0.003
Malignancies	0.000	–0.245	–0.244
Diabetes	0.176	–0.084	–0.071
Current smoker	0.236	0.151	0.079
Former smoker	–0.356*	–0.105	–0.206
More than 10 cigarettes	–0.235	–0.167	–0.256
No. of cigarettes	–0.166	–0.116	–0.277
Cigarettes — packs/year	–0.195	–0.150	–0.291
Allergies	0.386*	0.171	0.210
Postmenopausal since when	0.218	–0.049	–0.176
Gravidity	–0.130	0.008	–0.214
Parity	–0.111	0.158	–0.057
Miscarriage(s)	–0.195	–0.324	–0.493**
**Family history**			
Mother with painful periods	0.087	0.154	0.119
Mother had endometriosis	0.042	0.091	–0.069
Sister with painful periods	–0.004	0.071	0.071
Sister had endometriosis	0.250	–0.030	0.068
Daughter with painful periods	–0.149	0.028	–0.028

	Postmenopausal		
	**Pain intensity**	**Dyspareunia**	**Sexual dysfunction**
Daughter had endometriosis	–0.031	–0.304	–0.303
**Premenopausal symptoms**			
General condition	0.483**	0.248	0.244
Pain since first period (yes/no)	–0.125	–0.112	0.037
Pain intensity since first period	–0.165	–0.003	0.089
Pain (yes/no)	0.276	0.135	0.244
Pain intensity	–0.022	0.228	0.226
Pain during sexual intercourse	0.284	0.354*	0.287
Pain intensity during sexual intercourse	0.370*	0.385*	0.287
Rarely with pain during sexual intercourse	0.157	0.216	0.128
Always with pain during sexual intercourse	0.042	0.274	0.224
Pain during sexual intercourse only at ovulation	–0.018	0.049	0.048
Pain during sexual intercourse only before/after menses	0.143	–0.079	–0.123
Sexuality affected by endometriosis	0.385*	0.625**	0.607**
Intestinal cramp	0.160	0.217	0.243
Dyschezia	0.108	0.048	–0.012
Diarrhea	0.056	–0.029	0.083
Constipation	–0.217	–0.092	–0.213
Mucus in feces	0.228	0.039	0.090
Blood in feces	0.319	0.235	0.234
Dysuria	0.238	0.261	0.132
Blood in urine	0.151	0.075	0.138
Urinary stasis	–0.019	0.028	0.103
Increased urinary urgency	0.262	0.275	0.214
Incontinence	0.114	0.198	0.031
Retrosymphyseal pain	0.029	0.248	0.132
With strenuous activities	0.460**	0.359*	0.300
With moderately strenuous	0.516**	0.410*	0.287

	Postmenopausal		
	**Pain intensity**	**Dyspareunia**	**Sexual dysfunction**
activities			
Carrying shopping bag	0.461**	0.366*	0.341*
Climbing steps	0.453**	0.564**	0.362*
Climbing one step	0.353*	0.359*	0.306
Walking > 1 km	0.548**	0.496**	0.385*
Restrictions when bending/kneeling/stooping	0.643**	0.588**	0.401*
Restrictions when bathing/dressing	0.256	0.260	0.089
Physical restrictions	0.500**	0.427*	0.336*
Psychological problems	0.530**	0.514**	0.453**
Impairment of social contacts	0.500**	0.507**	0.506**
Impairment of everyday living/work	0.182	0.433**	0.496**
**Postmenopausal symptoms**			
General condition	0.641**	0.365*	0.403*
Pain (yes/no)	0.862**	0.430**	0.361*
Pain due to endometriosis	0.616**	0.554**	0.484**
Pain with other causes	0.080	–0.314	–0.235
Pain during sexual intercourse	0.468**	0.912**	0.730**
Intestinal cramp	0.467**	0.174	0.250
Dyschezia	0.313	0.253	0.304
Diarrhea	0.070	–0.116	–0.205
Constipation	0.200	0.056	0.125
Mucus in feces	0.216	–0.059	0.067
Blood in feces	0.255	0.197	0.303
Dysuria	0.409*	0.443**	0.482**
Urinary stasis	–0.192	–0.170	–0.170
Increased urinary urgency	0.396*	0.438**	0.367*
Incontinence	0.195	0.395*	0.421*
Retrosymphyseal pain	0.579**	0.623**	0.547**
Association with bladder symptoms	0.393*	0.633**	0.590**
With strenuous activities	0.553**	0.499**	0.402*
With moderately strenuous	0.596**	0.512**	0.501**

	Postmenopausal		
	**Pain intensity**	**Dyspareunia**	**Sexual dysfunction**
activities			
Carrying shopping bag	0.553**	0.396*	0.467**
Climbing steps	0.606**	0.389*	0.170
Climbing one step	0.516**	0.376*	0.293
Restrictions when bending/kneeling/stooping	0.658**	0.643**	0.456**
Walking > 1 km	0.644**	0.441**	0.349*
Restrictions when bathing/dressing	0.406*	0.092	0.031
Physical restrictions	0.676**	0.691**	0.605**
Physical restrictions of other cause	0.228	–0.059	–0.174
Psychological problems	0.471**	0.435**	0.407*
Psychological problems of other cause	0.030	–0.267	–0.307
Impairment of social contacts	0.164	0.264	0.293
Impairment of social contacts of other cause	–0.129	–0.238	–0.247
Impairment of everyday living/work	0.706**	0.459**	0.548**
Impairment of everyday living/work of other cause	0.280	–0.243	–0.194
**Premenopausal medication**			
Premenopausal medication (yes/no)	0.175	0.287	0.180
GnRH analogues	0.010	0.129	–0.112
Oral contraception	0.126	0.229	0.169
Period of medication	0.182	0.253	0.143
Success of medication	0.144	0.192	0.066
Side effects of medication	0.090	0.281	0.157
**Postmenopausal medication**			
Hormone replacement therapy	0.343*	0.485**	0.480**
Period of medication	0.276	0.463**	0.449**
Side effects of medication	0.348*	0.338*	0.337*
Alternative medicine treatments	0.059	0.228	0.348*

	Postmenopausal		
	**Pain intensity**	**Dyspareunia**	**Sexual dysfunction**
**Surgical treatment**			
Gynecological operations, total	0.168	0.142	0.070
Gynecological operations with endometriosis	0.213	0.221	0.120
Gynecological operations without endometriosis	0.013	–0.074	0.038
Laparoscopy	0.061	0.188	0.097
Laparotomy	0.215	0.128	0.089
Hysteroscopy	0.075	–0.245	0.013
Curettage	–0.118	–0.246	–0.267
Hysterectomy	0.320	0.150	0.201
Hysterectomy without adnexa	–0.335*	–0.284	–0.271
Hysterectomy with unilateral adnexa	0.278	–0.020	0.024
Hysterectomy with bilateral adnexa	0.412*	0.447**	0.445**
Time point of adnexectomy	0.229	0.299	0.303
Unilateral adnexectomy	0.278	–0.020	0.024
Bilateral adnexectomy	0.258	0.425*	0.424*

* $P < 0.05$

** $P < 0.01$

SUPPLEMENTAL DIGITAL CONTENT 2:

The following questionnaire items were used as independent variables for all multiple regression analyses:

- Difficulty when bending, kneeling or stooping (yes/no)
- Hysterectomy with bilateral adnexectomy (yes/no)
- Retrosymphyseal pain (yes/no)
- Blood in urine (yes/no)
- Pain during sexual intercourse (0–10)
- General pain due to endometriosis (0–10)
- Physical impairment due to endometriosis (yes/no)
- Drug intake for treatment of endometriosis or its symptoms (yes/no)
- Effects on sexual life due to endometriosis (0–10)
- Difficulty when climbing stairs (yes/no)
- Pain during defecation (yes/no)
- Side effects of drug intake (yes/no)
- Hysterectomy not including adnexa (yes/no)
- Impairment of everyday life or working life due to endometriosis (yes/no)
- Period of drug intake to treat endometriosis or its symptoms (months)
- Hysterectomy (yes/no)
- Hysterectomy with unilateral adnexectomy (yes/no)
- Urinary stasis (yes/no)
- Mother had painful menstruations (yes/no)
- Impaired social contacts (yes/no)

All of the above independent variables were related only to the premenopausal, active period of endometriosis.

Best regression model for predicting an effect on general postmenopausal pain due to premenopausal factors

	Regression coefficient B	*P* value	95% confidence intervals for B	
			Lower threshold	Upper threshold
(Intercept)	0.350	0.666	–1.296	1.997
Physical restriction when bending/kneeling/stooping	3.374	< 0.001	1.785	4.963
Hysterectomy with bilateral adnexectomy	3.549	< 0.001	2.235	4.863
Retrosymphyseal pain	–5.260	< 0.001	–6.921	–3.599
Hematuria	2.621	0.003	0.985	4.256
Pain during sexual intercourse INT	0.489	0.001	0.231	0.747
General pain INT	–0.331	0.006	–0.557	–0.105
Physical restriction	1.739	0.022	0.277	3.201
Hormone therapy	1.181	0.048	0.014	2.349

Model summary: R^2 = 0.833; P < 0.001.

Positive regression coefficient: increase in postmenopausal pain.

Negative regression coefficient: reduction in postmenopausal pain.

Best regression model for predicting an effect on postmenopausal pain during sexual intercourse due to premenopausal factors

	Regression coefficient B	*P* value	95% confidence intervals for B	
			Lower threshold	Upper threshold
(Intercept)	–2.194	0.001	–3.346	–1.042
Effect on sexual life	0.201	0.030	0.022	0.380
Hysterectomy with bilateral adnexectomy	3.533	< 0.001	2.481	4.584
Physical restriction when climbing steps	2.279	< 0.001	1.288	3.269
Dyschezia	–2.504	< 0.001	–3.592	–1.415
Drug side effects	1.979	0.001	0.847	3.111
Hysterectomy without adnexectomy	3.217	< 0.001	1.673	4.761
Impairment of everyday living and work	2.109	0.006	0.656	3.562
Medication (months)	–0.007	0.025	–0.012	–0.001

Model summary: R^2 = 0.857; P < 0.001.

Positive regression coefficient: increase in postmenopausal pain.

Negative regression coefficient: reduction in postmenopausal pain.

Best regression model for predicting impairment of postmenopausal sexuality due to premenopausal factors

	Regression coefficient B	*P* value	95% confidence intervals for B	
			Lower threshold	Upper threshold
(Intercept)	–4.163	< 0.001	–5.447	–2.880
Effect on sexual life	0.512	< 0.001	0.329	0.694
Dyschezia	–3.034	< 0.001	–4.224	–1.843
Hysterectomy	4.631	< 0.001	3.629	5.632
Hysterectomy with unilateral adnexectomy	–5.925	< 0.001	–7.629	–4.222
Impairment of everyday living and work	4.351	< 0.001	2.495	6.207
Urinary stasis	3.344	< 0.001	1.996	4.692
Medication (months)	–0.012	< 0.001	–0.018	–0.006
Drug side effects	1.773	0.007	0.532	3.013
Physical restrictions when climbing steps	2.986	< 0.001	1.726	4.247
Retrosymphyseal pain	–3.467	< 0.001	–5.144	–1.789
Dysmenorrhea in mother	2.290	0.004	0.833	3.747
Impairment of social contacts	–1.908	0.037	–3.693	–0.124

Model summary: R^2 = 0.931; P < 0.001.

Positive regression coefficient: increase in postmenopausal sexual impairment.

Negative regression coefficient: reduction in postmenopausal sexual impairment.
